# Mapping for Engagement: Setting up a Community Based Participatory Research Project to Reach Underserved Communities at Risk for Hepatitis C in Ho Chi Minh City, Vietnam

**DOI:** 10.3389/fpubh.2022.795470

**Published:** 2022-02-09

**Authors:** Giang Nguyen Quoc, My Nguyen Le Thao, An Bao, Ngoc Nguyen Anh, Vi Vu Thi Tuong, Diep Nguyen Thi Ngoc, Loc Phan, Thai Phan Minh, Thuy Lam Ngoc, An Nguyen Thanh, Thuan Nguyen Anh, Trang Nguyen Nguyen Nhu, Lan Nguyen Thi, Vy Nguyen Thuy Thanh, Hieu Nguyen Minh, Thuan Nguyen Minh, My Do Thuy An, Thong Nguyen Tri, Phung Tran Thi, Son Pham Hong, Ngoc Tran Thi, Anh Hoang Thai, Hanh Duong Thi My, Graham S. Cooke, Mary Chambers, Jennifer Ilo Van Nuil

**Affiliations:** ^1^Oxford University Clinical Research Unit, Ho Chi Minh City, Vietnam; ^2^CBPR Stakeholder Working Group, Ho Chi Minh City, Vietnam; ^3^CBPR Community Advisory Group, Ho Chi Minh City, Vietnam; ^4^Department of Infectious Diseases, Imperial College London, London, United Kingdom; ^5^Centre for Topical Medicine and Global Health, Nuffield Department of Medicine, University of Oxford, Oxford, United Kingdom

**Keywords:** stakeholder mapping, community-based participatory, community research engagement, hepatitis C (HCV), Vietnam, underserved populations

## Abstract

**Background:**

Approximately 1. 07 million people in Vietnam are infected with hepatitis C virus (HCV). To address this epidemic, the South East Asian Research Collaborative in Hepatitis (SEARCH) launched a 600-patient cohort study and two clinical trials, both investigating shortened treatment strategies for chronic HCV infection with direct-acting antiviral drugs. We conducted ethnographic research with a subset of trial participants and found that the majority were aware of HCV infection and its implications and were motivated to seek treatment. However, people who inject drugs (PWID), and other groups at risk for HCV were under-represented, although injecting drug use is associated with high rates of HCV.

**Material and Methods:**

We designed a community-based participatory research (CBPR) study to engage in dialogues surrounding HCV and other community-prioritized health issues with underserved groups at risk for HCV in Ho Chi Minh City. The project consists of three phases: situation analysis, CBPR implementation, and dissemination. In this paper, we describe the results of the first phase (i.e., the situation analysis) in which we conducted desk research and organized stakeholder mapping meetings with representatives from local non-government and community-based organizations where we used participatory research methods to identify and analyze key stakeholders working with underserved populations.

**Results:**

Twenty six institutions or groups working with the key underserved populations were identified. Insights about the challenges and dynamics of underserved communities were also gathered. Two working groups made up of representatives from the NGO and CBO level were formed.

**Discussion:**

Using the information provided by local key stakeholders to shape the project has helped us to build solid relationships, give the groups a sense of ownership from the early stages, and made the project more context specific. These steps are not only important preliminary steps for participatory studies but also for other research that takes place within the communities.

## Introduction

Viral hepatitis is a global health issue needing urgent attention. Globally, it is estimated that 257 million people are living with hepatitis B virus (HBV) (1) and 71.1 million people with hepatitis C virus (HCV) infection (2, 3). Low- and middle-income countries (LMIC) are thought to carry more than 80% of the HCV burden and Vietnam has one of the highest rates of mortality from chronic viral hepatitis deaths, alongside China and Japan (4). Approximately 1.07 million people in Vietnam are living with HCV (3). With the development and rollout of highly effective direct-acting antiviral treatment in 2015, HCV can now be cured and the possibility of elimination of HCV as a major health threat by 2030, a World Health Organization's (WHO) goal, is now a possibility (5). However, if people living with HCV are not aware of their status or they do not have access to treatment, it will be difficult to achieve.

To address this epidemic, the South East Asian Research Collaborative in Hepatitis (SEARCH) launched a 600-patient cohort study and two clinical trials, both investigating shortened treatment strategies for chronic HCV infection with direct-acting antiviral drugs. These studies have primarily recruited from populations already engaged in care at the Hospital for Tropical Diseases (HTD) in Ho Chi Minh City (HCMC), Vietnam. We conducted ethnographic research with a subset of the trial participants and found that the majority were aware of HCV infection and its implications, and were motivated to seek treatment. The absence of certain at-risk communities from the trial population was apparent. Overall, people who inject drugs (PWID), and other groups at risk for HCV were under-represented, although injecting drug use is associated with high rates of HCV, with an estimated 50–90% of PWID in Vietnam having HCV (6–8). Another group that is disproportionately affected by viral hepatitis are men who have sex with men (MSM). In Vietnam, it is estimated that 36.3–41.2% % of MSM have HCV (3, 6, 9). In healthcare settings, the seroprevalence of dialysis patients was found to be as high as 26.6% (3, 6), although these patients are likely accessing care and treatment. Transmission of HCV in Vietnam is thought to be caused mostly by unsafe intravenous practices, such as injecting drugs or blood-transfusions (3, 6, 10).

We had several questions about the potential underserved populations: who are the underserved populations at risk for viral hepatitis? Are there specific barriers to care? Are people engaged in care elsewhere? What can be done to improve linkages to testing, diagnosis, care, and treatment (if needed)? To explore these questions, we designed a study using community-based participatory research (CBPR) with an overarching aim to engage with communities at risk for viral hepatitis in order to develop community-led strategies to improve linkages to care and treatment. The main principles of CBPR are to build collaborative partnerships between an academic institute (in our case OUCRU), and community-based organizations (CBOs) (11). In CBPR approaches, the community members are involved in all aspects of the project from identifying the research problems, to developing and implementing community-led solutions that build upon the strengths and structures that already exist in the communities (11). When we first envisioned the project, we did not have direct links with relevant CBOs, nor were we fully aware of the resources already existing in the community, or the dynamics between key players within the communities. Therefore, we designed a preliminary phase of the project to focus on learning more about the community dynamics through stakeholder mapping, as well as to form stakeholder groups to advise us throughout the project more broadly.

Stakeholder mapping, as a method, can be useful for identifying and describing the relevant organizations and individuals from the communities who potentially influence decision making and have some working role with the communities (12, 13). Stakeholder mapping can result in several benefits: to assess the capacity of communities, to provide the community with an overview of potential resources, to create a visualization of the individuals and organizations that could influence, support, and help to solve community problems, and to demonstrate relationships and roles of various stakeholders within the communities (14–16). All of these outputs would be useful for the wider project. Fostering involvement and collaboration with stakeholders at various levels is crucial to CBPR projects. As “equal partners” in the relationship and in the project, the involvement of the stakeholders can help to create a more locally driven research focus based on the community's prioritized concerns (13). Involving stakeholder groups can also help to define appropriate research methods and culturally sensitive ways to approach and work with underserved groups (15). Additionally, stakeholder groups can also contribute credibility to the project and promote a higher chance of acceptability from local communities (15).

In this manuscript, we describe the first phase of the study which was set up to identify general characteristics of different underserved groups, their prioritized needs, as well as their potentialities and existing resources. The description of this phase is often limited in other articles using similar approaches and we find it a crucial component. In this article, we describe and discuss the preliminary phase, not only as a preparatory step before implementing the main study, but also as an essential starting point of the CBPR process. The process of mapping stakeholders initiated the partnership process with non-governmental organizations (NGOs) and CBOs, creating the necessary linkages between OUCRU researchers and underserved community groups, as well as helped to shape the research questions for the broader project.

## Materials and Methods

We used a CBPR approach following the principles described by Israel (1998). Our particular interest was to explore the local perceptions surrounding viral hepatitis, barriers to care seeking, as well as learn more about the strengths and structures of the communities in which we would be working. This phase included stakeholder mapping meetings with advisory groups and the formation of two stakeholder working groups. The goal of this phase was to identify key stakeholders or groups/organizations working with underserved people at risk for HCV in and around HCMC, Vietnam.

### Advisory Groups for Stakeholder Mapping

**Figure 1:**
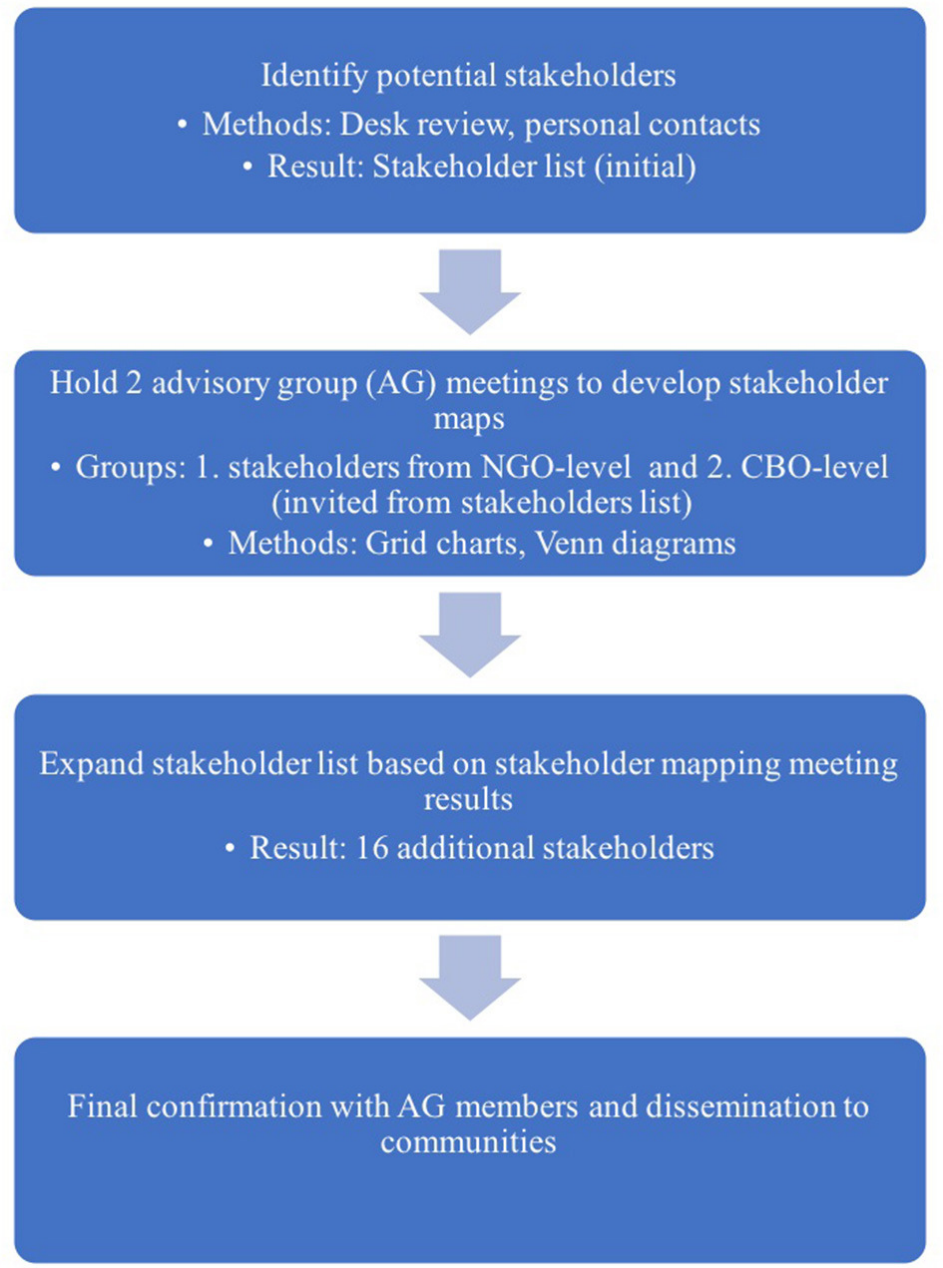
Steps conducted for stakeholder mapping.

We started the stakeholder mapping by conducting a desk review to identify stakeholders working with potentially underserved populations at risk for HCV in HCMC. We used personal contacts to create an initial list of key stakeholders including representatives from various organizations with an interest in viral hepatitis and/or working with underserved communities, and invited them to attend one of two advisory groups (AGs) meetings, one at the NGO-level and the second at the CBO-level. The goal of the meetings was to conduct stakeholder mapping using two main tools: grid charts and Venn diagrams. We used grid charts to summarize information and identify additional NGOs, CBOs, and informal groups working with underserved populations to expand the initial desk review mapping exercise (16). We then used Venn diagrams to summarize and illustrate the perceived connections, influences, and relationships among and between the stakeholders and key populations. We also hoped that these diagrams would potentially show where and how to gain access to and cooperate with community stakeholders (17). See Figure 1.

### Formation of SWGs

From the individuals who attended the AGs mapping meetings, we formed two stakeholder working groups (SWGs) to collaborate and advise us throughout the CBPR process. Before creating the groups, we discussed the SWG roles, commitments and approximate timing for meetings and activities. Additionally, we discussed the voluntariness of joining the SWG, the CBPR groups and the ways we could maximize confidentially within the project more broadly.

### Ethical Considerations

Throughout the preliminary phase, two researchers from OUCRU observed and wrote fieldnotes on the process and content of the meetings. At the start of each meeting, the researchers made it clear to all participants that the meeting dialogues would be documented by written notes and we obtained verbal consent to take photos during the meeting. All potentially confidential data from SWG meetings would only be shared between SWGs members and the research team. This manuscript is based on the discussions within the meetings held by the two SWGs and has been co-produced with them. The full study was approved by Oxford Tropical Research Ethics Committee (OxTREC) at University of Oxford (OxTREC 556-20), Imperial College Ethics Committee (20IC6420) and locally by the CBOs under which the CBPR groups are formed.

## Results

### Results of Mapping Meetings

Based on the desk review and input from the initial stakeholders that we contacted, we held two meetings with representatives from two broad groups: those working at the NGO and CBO levels, to explore a range of perspectives. The contacts informed us that it would be better to separate the AGs into these broad categories for enhanced participation, especially for the CBO-level. We invited relevant participants from NGOs, CBOs, the private sector, and community clinics to join the AG meetings. The AG meeting with the NGO representatives took place on 1st September 2020 with 13 participants and the AG meeting for the CBOs took place on 01st October 2020 with 16 participants. Both meetings lasted approximately two and a half hours (see Table 1).

**Table 1:** Participant-types in AG stakeholder mapping meetings.

**Stakeholder types**	**Number of** **organizations**	**Number of** **participants**
**NGO-level AG meeting**		
NGO	5	10
Institute	1	2
Private sector	1	1
**CBO-level AG meeting**		
CBO*	6	13
Community clinic	3	3

* *3 of the CBOs were also social enterprises*.

Overall, the individuals who participated in the meetings had experience working with vulnerable communities including MSMTG, PWID, HIV, sex workers and those in poverty. Members from NGOs had experience in consulting and providing technical assistance, capacity building for community organizations related to the implementation of prevention and treatment programs on HIV, STIs, nutrition and other issues. They also played a role in connecting and introducing community organizations to potential donors and funding mechanisms. Within the CBO groups, the leaders were mainly members from within those communities and therefore understood their contexts and needs. In some instances, participants from the CBOs also considered themselves members of the underserved communities. The CBOs conducted outreach to those affected by various diseases, such as HIV, sexually transmitted infections (STIs), HBV and HCV, provided access to health screenings and linkage to care, as appropriate. According to participants from both meetings, the CBOs had close working relationships with members of the community as well as with the organizations providing health services.

In each AG meeting, the participants were asked to conduct stakeholder mapping using grid charts and Venn diagrams. To conduct these activities, we divided them randomly into two smaller groups to facilitate more discussion and build consensus on the key stakeholders working within this realm.

### Creating Grid Charts

The stakeholder grid was designed to include the NGO name, year established, funding resources, key populations, and the main projects or activities being conducted. In each AG meeting, the participants created grid charts and selected one member to present the results to the wider group.

In the NGO-level meeting, the participants followed the grid chart template and listed the information as requested. Overall, they listed 15 organizations and other groups working in the communities, including six organizations that were not on the initial desk review mapping list. It also became an opportunity for the participants to introduce groups they knew and/or were part of and learn about each other's organizations. At the end of the exercise, we asked participants to review the draft stakeholders list that the OUCRU team had previously made. They were asked to validate or edit NGOs/CBOs' locations, contact information and program that they were conducting.

**Figure 2:**
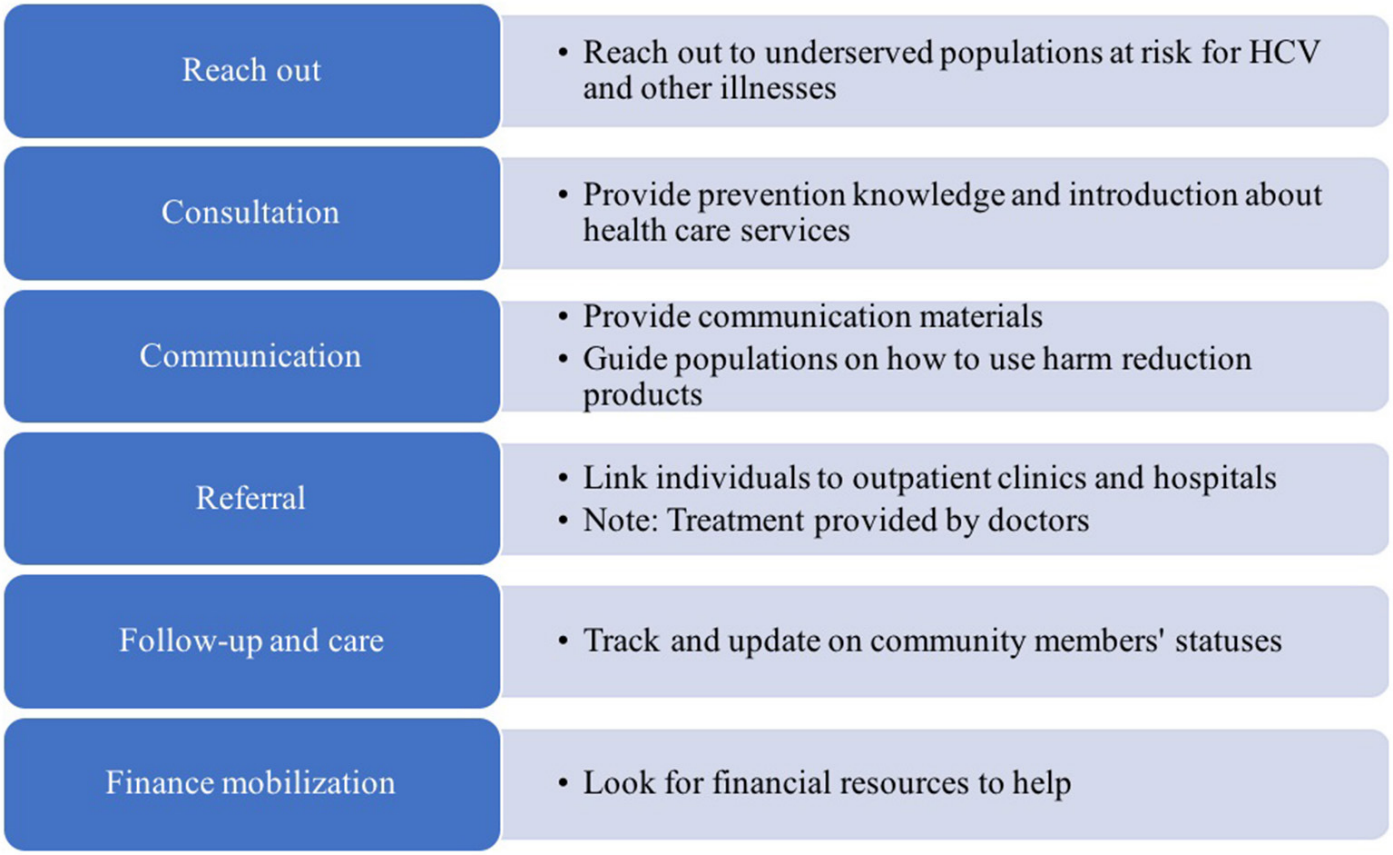
Stakeholder activities, as defined in the CBO-level AG meeting.

During the CBO-level meeting, the participants also divided into two smaller groups to create the grid charts. The first group introduced the key stakeholders by explaining the steps of the process typically used to support underserved community members. Each step described supporting activities, as well as the roles of CBOs and other related stakeholders in those activities (see Figure 2). The second group provided a list of stakeholders that had experiences in supporting people with HCV in underserved communities. This exercise contributed updated information about the activities and the background of community stakeholders than the draft that we originally summarized.

We observed two main differences between the NGO-level and CBO-level AG approaches to completing the grid charts. Firstly, in the NGO-level meeting, the participants listed mostly larger organizations that focused on providing funds and technical support to the local organizations; whereas in the CBO-level meeting, participants identified organizations receiving the funds and technical support. Secondly, in the CBO-level meeting, the participants revealed several challenges they encountered when providing services within their communities, and they also identified stakeholders' roles during the different implementing steps for typical activities. For example, if a CBO is involved in all the steps from identifying people at risk of HCV to referring them to treatment, then during the step “referring to treatment,” the CBO AG also added the information that “the CBO would collaborate with a clinic/hospital that provides the treatment.” At this stage, there is involvement from other stakeholders (e.g., clinic, hospital).

Overall, in the two mapping meetings, we identified 26 institutions or groups working with the key populations, including 16 institutions that were not on our original mapping list. The results of the stakeholder mapping were made available for the CBOs and the wider communities.

### Developing Venn Diagrams

**Figure 3:**
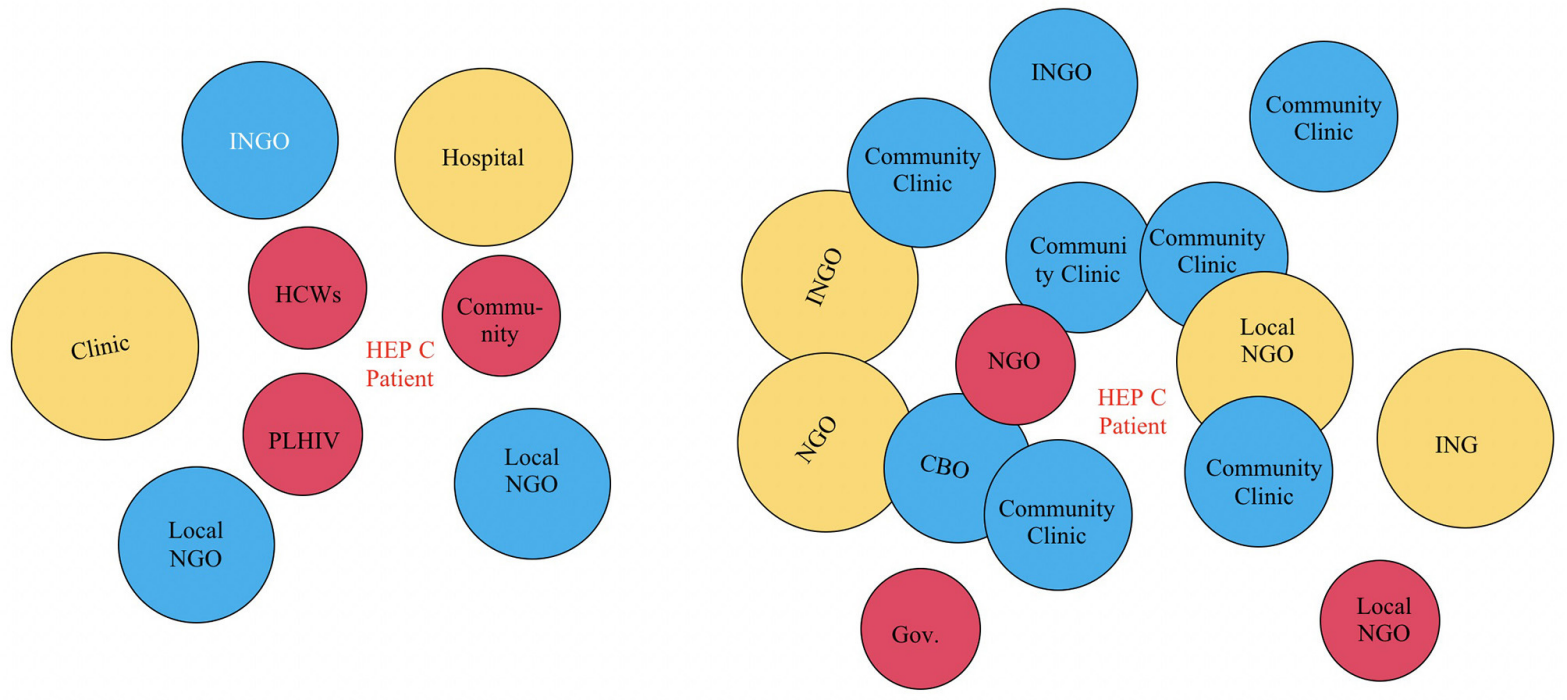
Venn diagrams from NGO-level AG meeting. The circle size implies perceived level of influence from the stakeholder to the center. The distance from the circle to the center point implies access level from the stakeholder to the center. HCWs, Healthcare workers; PLHIV, People living with HIV; CBO, Community Based Organization; INGO, International Non-Government Organization; Local NGO, Local Non-Government Organization.

The next part of the meetings was to create Venn diagrams from the lists created during the grid chart exercise. In the NGO-level meeting, the influence of stakeholders was divided into different categories (i.e., research, consultations, linkage to care and treatment) and the duration of influence was determined by the category of influence (e.g., research: when a research project lasts for only 2 years, the influence reduces after the project is completed; consultation/raising awareness: might be longer-term engagement and therefore have longer-lasting influence). A few concerns were raised by the NGO AG during this part of the exercise. According to the participants, the Venn diagram is subjective and potentially biased because representatives from the CBOs who were included in the diagram were not present at the meeting and could not contribute their perceptions. Second, the participants recognized that they analyzed their organizations from their perspectives only. Regarding the level of influence of organizations, the participants also mentioned that it was important to note that there are different types of influences, (e.g., influences regarding research, diagnosis, and/or consultancy) and the duration of such influences varies dramatically. See Figure 3.

**Figure 4:**
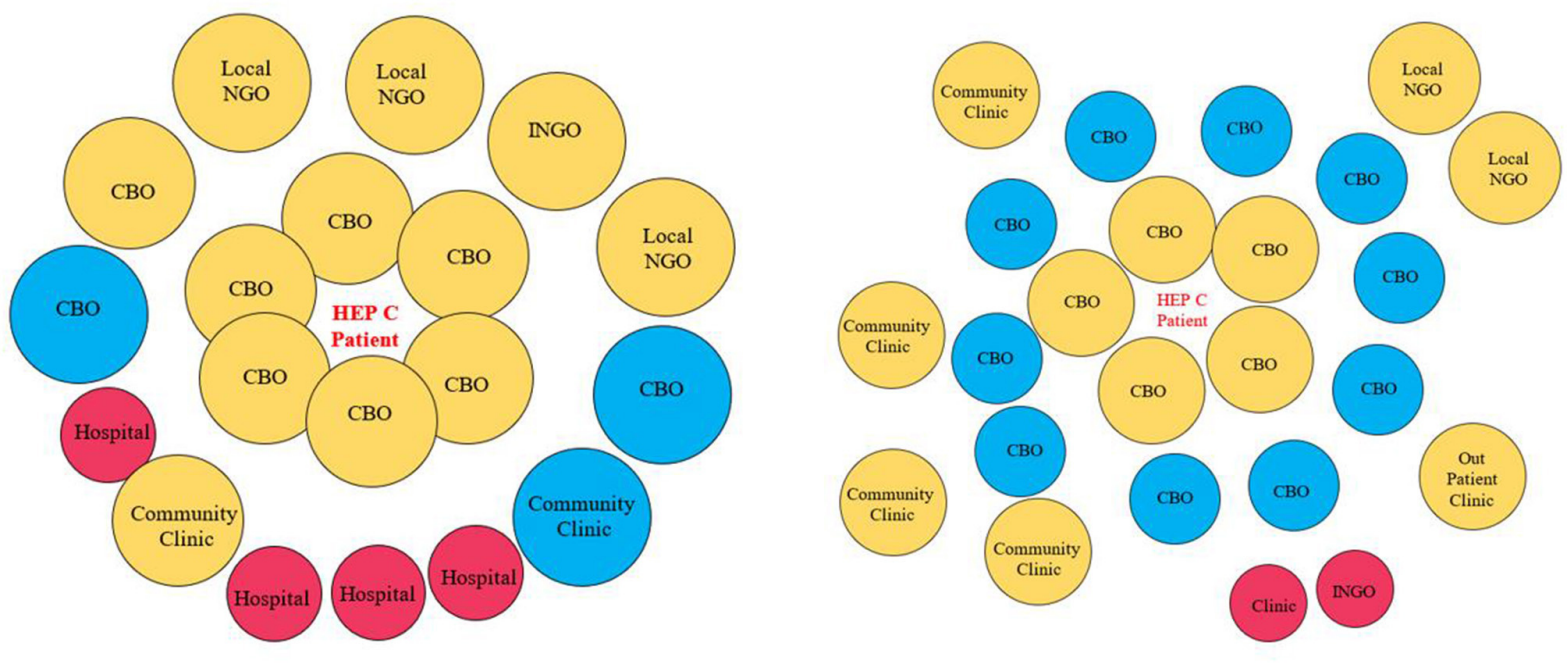
Venn diagrams from CBO-level AG meeting. The circle size implies perceived level of influence from the stakeholder to the center. The distance from the circle to the center point implies access level from the stakeholder to the center. CBO, Community Based Organization; INGO, International Non-Government Organization; Local NGO, Local Non-Government Organization.

In the CBO-level meeting, the group discussed that the larger institutions, with the potential for more influence, were not always embedded in the community, unlike the CBOs which tended to be embedded within the community. One group gave the example of an international NGO with a variety of projects in the community. Although this organization may have a big influence on communities, they were considered “far from the center” because they do not work directly in the community and therefore were less accessible to the community. The CBOs were “closer to the center” and were typically more accessible. Another point brought up in the CBO-level meeting was that if one changes the middle point of focus (e.g., in the meeting, it was HCV), everything around it changes as well so these dynamics are in constant flux. See Figure 4.

The priorities of the stakeholders also determined how the dynamics played out in the community and for specific key populations. For example, the CBO-level participants also discussed a “rupture” in the context of linking patients to treatment. There was a past research team that set up consultation and screening but did not link potential participants to care upon diagnosis. The research team returned to the community months later and asked what people had done with their diagnosis since that time, which was nothing as they did not know where to go for care. The participants felt lost as they were left with a diagnosis but not given advice about what to do with it. “Rupture” often happened when the research or project's aim was solely about screening but not about linking to treatment or longer-term follow-up. When the CBOs connected with the community after this happened, they found that the community did not want to engage with that institution/research team anymore—the relationship was “ruptured.”

For the Venn diagram exercise, the NGO-level and CBO-level participants had different perceptions regarding the influences and relationships of the institutions. For example, the NGO-level participants listed only one CBO on their diagram and placed it far from the underserved populations who were located in the center of the diagram. They placed clinics and hospitals closer to the underserved populations. On the other hand, the CBO-level participants placed the CBOs very close to HCV (which was in the center) and clinics and hospitals further away. Interestingly, the CBO group placed the disease at the center, not the key underserved populations explaining that if the disease changes (e.g., from HCV to HIV), then the dynamics surrounding it would also change. Additional key differences between the NGO-level and CBO-level AG approaches to the Venn diagram exercise are included in Table 2.

**Table 2:** Key differences between the NGO-level and CBO-level regarding key stakeholders in community, based on Venn diagram.

**NGO-level AG meeting**	**CBO-level AG meeting**
Community clinics are closest to the key populations (i.e., at the center) which means the populations find it easier to access community clinics than the other stakeholders/institutions included. Stakeholders/institutions that have the highest levels of influence are not always the organizations that are easy for key populations to access.	The CBOs that work directly with key populations (e.g., PWID, sex workers, HIV) have the highest influence and are closer to the communities (i.e., easier for the community to access). There are other stakeholders who have equally high influence but are not that close to center because they are not easy for key populations to access. The community clinics also have high influence but are not as close to key populations because they do not work only with key populations (e.g., transgender, MSM). The public hospitals are quite far from center with less influence noted than the other stakeholders.

### Results on Forming Stakeholder Working Groups

After the AG meetings, we formed two stakeholder working groups (SWGs) made up of representatives from the AG meetings. We invited all the participants from the initial AG meetings to join the two SWGs. We also held the first meetings with each group. During these initial meetings, we defined the roles and responsibilities for each group, discussed the advantages and disadvantages of using CBPR to make sure it was the appropriate approach for the project, explored the exact groups that made up “underserved” groups (with the NGO-level SWG) and identified specific groups with whom we could work (with the CBO-level SWG). Within the CBO-level SWG, we also identified community activators (CAs) who would be instrumental in setting up and leading the CBPR groups.

### Roles and Responsibilities

Before the initial SWG meetings, the research team drafted the terms of reference for each SGW, which described the scope of work, roles, benefits, and other necessary terms, and distributed them to the members prior to the meeting to start the discussions. At the start of the meeting, the research team asked the participants to define how they envisioned their roles in the study. We all agreed that the NGO-level SWG would be more of an advisory group while the CBO-level SWG would act more as community consultants. The exact roles discussed and defined by the groups are listed in Table 3.

**Table 3:** Roles of SWGs: NGO-level and CBO-level.

**NGO-level SWG**	**CBO-level SWG**
Orient CBPR groups on various ways to work with the communities. Provide overarching technical support. Provide suggestions on selection of sites and participant recruitment strategies.	Lead the CBPR groups, as CAs. Plan for research activities that take place in CBPR groups. Refer people to the HCV treatment trial, as appropriate.

### Advantages and Disadvantages of CBPR

Before discussing the strengths and weaknesses in the CBPR approach, we also discussed the approach itself. There were differences between what the groups thought about the core principles of CBPR. The NGO-level SWG focused on principles to use during the conduct of CBPR, including honesty, respect, equality, flexibility, and with the focus on the participants. For the CBO-level SWG, the emphasis centered more on the consequences of CBPR for participants (e.g., confidentiality, dedication to helping all, non-discrimination), and the importance of understanding the needs of each situation using two-way communication and evidence-based solutions.

The NGO-level SWG and CBO-level SWG also had different perspectives regarding the strengths and challenges of implementing CBPR in the community. The NGO-level SWG felt that the strengths of the approach included gaining data from multiple viewpoints, flexibility, and the aspects about building trust that would be enhanced. However, they also felt that there could be conflicts between members, there might be too much information gathered, and the NGO-level SWG and research team might have different long term expectations. The CBO-level SWG discussed the strengths of the approach including aspects surrounding how the CBOs are integrated within the communities, therefore could collaborate well with the CBPR groups (e.g., they understand the realities of the populations and there is pre-existing trust). They also thought that the methods, although new, would provide a diversity of information, reach more people, and the underserved populations would be easy (for them) to approach and collect data. Some of the challenges the CBO-level SWG discussed included lack of facilitation skills, information overload or misinterpretation of data, the workload and costs might be too much for the CBPR groups, and there may be a lack of trust in the community toward researchers, and/or different expectations and levels of commitment from the CBPR group members. In the end, both groups agreed that CBPR was the appropriate approach for answering the broader research questions.

### Identification of Underserved Populations and Groups to Work With, and CAs

At the NGO-level SWG meeting, the OUCRU research team suggested that the potentially underserved populations included PWID, sex workers, MSMTG, and people living with HIV. The NGO-level SWG members identified an additional at-risk group affected by HCV which included those who have low-income, unsustainable employment, and financial barriers to access regular care and treatment.

During the CBO-level SWG meeting, we invited members to volunteer as CAs to coordinate the CBPR groups and mobilize community members to participate. The role of the CAs was crucial for inviting members to join the group, to support group members during meetings, and to collect and analyze data together with the members of the groups. Each person at the CBO-level SWG meeting was given a card and if they wished to be a CA for a CBPR group, they simply wrote “Yes” on the card (with their name and contact), otherwise, they could leave the card blank. The project team compiled the list and responded to the individual members *via* email to confirm. In the end, each group had at least two CAs appointed by the CBO-level SWG.

### Community Activator Trainings

As requested by the CBO-level SWG, the OUCRU team organized training activities to equip the CAs with more knowledge about CBPR background and methods. We held a two-day training on CBPR, which focused on general definitions and principles of CBPR, and introduction to some of the basic tools of participatory research (e.g., Venn diagrams, grid charts, body mapping), as well as facilitation skills. Twelve participants attended the training as we opened it up to other interested participants from the SWGs. One of the most important aspects of this training was to stress how CBPR should be based on the issues of the community and how it is the community members who should decide the solutions for those problem. Listening and respecting differences was key.

## Discussion

The importance of understanding and listening to experts in the communities in which we work cannot be overstated. However, taking a step back and trying to understand the range and scope of expertise that already existed in the community was equally important for developing a dynamic within the already well-established community of stakeholders. The mapping exercise, along with the mapping meetings allowed us to achieve this goal. The subsequent dialogues and engagement with potential community leaders were crucial to the success of the development of the project as we had not worked with these communities in the past and we wanted to build the project with the communities from the start.

In our case, keeping the mapping methods fluid resulted in the CBO-level AG group transforming the method into a more informative and applicable method for the purposes of the exercise. With their tailored method, we learned about some of the challenges in implementing public health programs and individual care seeking in those communities. We also noted how the perceptions and priorities were different between the NGO- and CBO-levels working with the same communities. This minor point speaks volumes about the importance of listening and identifying who represents the community and how their actions potentially impact that perception. We intend to also compare these findings with the CBPR groups as their perceptions and priorities might also be different from the organizations that “represent” them.

The stakeholder mapping also provided the initial space for the researchers to start to understand the potential strengths and resources of the community, as it was clear that there was indeed a community prior to the start of this project. From an outsider perspective, it seemed like the stakeholders involved in the meetings were already part of a close-knit community. We also noted quickly how different forms of organizations have different roles in the community. An outcome of the mapping meetings was the fact that the communities themselves were able to start to advise researchers prior to the study officially beginning. These initial meetings set the tone for the future participatory work. Mathur et al. (18) discussed how stakeholder mapping can be a complex technique, but it can be an effective way to better understand stakeholders, their influences on each other, and for assessing the research topics at hand. During the discussions in the stakeholder mapping meetings, the participants from both the NGO-level and CBO-level AGs spoke about the relationships among the stakeholders and their ability to influence the health issues that underserved groups potentially faced, as well as listed out services were provided for these populations in their communities. In reality, most NGOs were involved in policy advocacy, implemented fundraising activities with both local and international stakeholders, and managed and allocated funds to CBOs in specific priority areas. The CBOs worked directly with community members to provide consultations, linkages to screening, treatment, and follow-up care. As an initial exercise and first meeting together, it was useful to start to understand the dynamics of the stakeholders and how they worked within the communities.

One important aspect of the CBPR approach is to create equal partnerships. Participatory stakeholder mapping can be used as a first step to create a shared research environment for community members with more balanced roles prior to implementing CBPR or other community-focused research, as this balance of power dynamics between the researchers and community members, or even between community members is often difficult to achieve (15, 19). In a study conducted by Kue et al. (15) with Hmong communities in the United States, community-based methods were used as well as a community advisory committee formed to provide insights to the communities' social patterns and resources to define culturally appropriate data collection methods. The members of this committee ranged in age and gender but younger members and women were observed to be less active in discussions compared to older and male members because, according to the researchers, the roles (and voices) of these members in the community were already defined (15). The cultural norms played a role in making equal or balanced participation difficult to achieve. With this in mind, while we were setting up the initial AG mapping meetings, we consulted with a selection of stakeholders prior to the meetings and decided together to divide the groups in NGO- and CBO-levels because of the power dynamics that already existed in these communities.

Finally, our assumptions about who was at risk for HCV was missing a key group—the financially vulnerable communities, and by holding these early conversations we were able to expand the research groups for inclusion in the project beyond those that we had planned. Our initial impressions of the communities were incomplete.

One limitation of this paper is that we only present the results from the preliminary phase of the project, however the details of how the CBPR groups formed and progressed will be presented elsewhere. A second limitation is that the majority of the meetings described in this paper were conducted in Vietnamese and therefore some meaning may have been lost in translation into the English version. Third, although the CBO-level participants who were engaged in this stage of the project were working directly with and were sometimes community members themselves, their views and opinions may be different than community members who were not directly involved with the CBOs. In future studies, it may be worth adding a third group including only those from underserved communities to determine how their opinions might differ at this stage.

To conclude, listening early, carefully, and often has helped us to build solid relationships. Using information generated by the community to shape the project has provided a mutual sense of ownership from the early stages of the project and also created a more context specific project. These initial steps are not only important preliminary steps for participatory studies but also for other research that takes place within the communities. The methods allowed all involved to consider their own approaches and activities within the communities and plan for a more collaborative and participant-led initiative.

## Data Availability Statement

The raw data supporting the conclusions of this article will be made available by the authors, without undue reservation.

## Ethics Statement

The studies involving human participants were reviewed and approved by Oxford Tropical Research Ethics Committee (OxTREC) at University of Oxford (Ref: OxTREC 556-20) Imperial College Ethics Committee (Ref: 20IC6420). The CBPR participants provided their written consent to participate in the study.

## Author Contributions

JV, MC, and GC initially designed the main study and obtained funding. GN, MN, AB, NN, VV, DN, LP, TP, TL, AN, TNA, TNNN, LN, VN, HN, TNM, MD, TNT, PT, SP, NT, AH, and HD provided input into the development of the project and were involved in the preliminary phase of the study. JV and GN prepared the manuscript draft, with input from MC and MN. All authors reviewed and approved the final manuscripts.

## Funding

This work would not have been possible without funding from the Wellcome Research Enrichment Award 206296/Z/17/A. For the purpose of open access, the authors have applied a CC BY public copyright license to any Author Accepted manuscript version arising from this submission. GC is supported by an NIHR Professorship and the NIHR Biomedical Research Centre of Imperial college.

## Conflict of Interest

The authors declare that the research was conducted in the absence of any commercial or financial relationships that could be construed as a potential conflict of interest.

## Publisher's Note

All claims expressed in this article are solely those of the authors and do not necessarily represent those of their affiliated organizations, or those of the publisher, the editors and the reviewers. Any product that may be evaluated in this article, or claim that may be made by its manufacturer, is not guaranteed or endorsed by the publisher.
